# A rare case report of renal ewing sarcoma/primitive neuroectodermal tumor with ACTH production

**DOI:** 10.1186/s12894-022-01055-y

**Published:** 2022-07-11

**Authors:** Weipu Mao, Jiajia Xu, Haowen Lu, Yali Wang, Lihua Zhang, Ming Chen

**Affiliations:** 1 Department of Urology, People’s Hospital of Putuo District, No.1291, Jiangning Road, Putuo District, Shanghai, 200060 China; 2 Department of Urology, Affiliated Zhongda Hospital of Southeast University, Nanjing, 210009 China; 3 Department of Urology, Nanjing Lishui District People’s Hospital, Zhongda Hospital Lishui Branch, Southeast University, Nanjing, 211200 China; 4 Department of Pathology, Affiliated Zhongda Hospital of Southeast University, No. 87 Dingjiaqiao, Hunan Road, Gulou District, Nanjing, 210009 China

**Keywords:** Ewing sarcoma/primitive neuroectodermal tumor, Cushing syndrome, Hypothyroidism, EWSR1, Case report

## Abstract

**Background:**

Ewing sarcoma/primitive neuroectodermal tumor (PNET) of the renal is extremely rare. The common cause of ectopic ACTH syndrome is pulmonary neuroendocrine tumors, such as small cell carcinomas and carcinoid tumors. Here, we present an unusual case of ectopic ACTH syndrome and hypothyroidism caused by Ewing sarcoma/PNET of the right kidney.

**Case presentation:**

A 19-year-old girl presented with a history of right lumbar pain and discomfort for 2 months, aggravated for 2 days. Abdominal contrast-enhanced computed tomography and computed tomography angiography showed an upper pole occupancy of the right kidney occupancy with subepithelial hemorrhage. Preoperative hormone levels including plasma total cortisol (PTC), adrenocorticotrophic hormone (ACTH) and thyroid hormone measurements were abnormal, indicating that the patient had Cushing syndrome and hypothyroidism. The patient underwent right radical nephrectomy. Histopathological analysis revealed a renal small round blue cell tumor (consistent with a primitive neuroectodermal tumor), with positive immunohistochemistry for CD99 and Ki67 (about 10%) and molecular pathology for EWSR1 gene fusions. PTC, ACTH and thyroid hormone returned to normal after surgery.

**Conclusions:**

We report a rare ectopic ACTH syndrome and hypothyroidism due to renal Ewing sarcoma/PNET. The clinical manifestation of renal Ewing sarcoma/PNET is non-specific and the diagnosis relies on pathological morphology, immunohistochemistry and fusion gene detection. At present, surgery combined with radiotherapy and chemotherapy is used in the treatment, but the prognosis is still not optimistic.

## Background

Ewing sarcoma/primitive neuroectodermal tumor is a family of small round blue cell malignancies including bone and soft tissue Ewing sarcoma, Askin's tumor and primitive neuroectodermal tumor (PNET). It occurs mostly in the long bones and pelvis, and rarely in the kidney [1, 2]. Small round blue cell tumors are histologically characterized by proliferation of small round tumor cells with sparse cytoplasm, which are often difficult to distinguish by standard histology or immunohistochemistry [3, 4]. The disease usually has an insidious onset, rapid progress, easy recurrence and metastasis, and poor prognosis. Patients with Ewing sarcoma/PNET require rapid treatment [5].

Cushing syndrome caused by excessive secretion of ACTH from tumor tissues other than the pituitary gland is called ectopic ACTH syndrome, accounting for 10–20% of Cushing syndrome [6]. The common causes of ectopic ACTH syndrome are lung or bronchial tumors, followed by thymic and pancreatic tumors [7]. Herein, we introduce a rare case of ectopic Cushing syndrome and hypothyroidism caused by a malignant tumor of Ewing sarcoma/PNET in the right kidney.

## Case presentation

A 19-year-old girl presented to our hospital with a history of right lumbar pain and discomfort for 2 months and aggravated for 2 days. The patient felt the right waist pain 2 months ago, which was persistent dull pain, and increased right-sided back pain after running 2 days ago. Abdominal computed tomography (CT) showed right renal hamartoma with hemorrhage in the local hospital. The patient had no significant medical history and was not receiving any medication at the time of referral. Of note in the history was that the patient discovered facial swelling, increased acne on the face and back, and scanty menstruation six months ago. Upon admission, physical examination revealed concentric obesity and positive percussion pain in the right costal horn.

### Laboratory examination

Routine blood tests showed elevated infection indicators, abnormal renal and liver function, elevated thyroid hormone and female hormone levels, and abnormal cortisol rhythm and adrenocorticotropic hormone (ACTH) rhythm (Table 1).

**Table 1:** Changes in patients' laboratory examination results

Characteristics	Normal ranges	Sep 5	Sep 6	Sep 7	Sep 8 (8:00 am)	Sep 8 (5:00 pm)	Sep 9	Sep 10	Sep 11	Sep 21
*Blood routine*										
White-cell (10^9^/L)	3.5–9.5	17.15	24.1		19.87	26.74	11.36	8.89	6.69	7.41
Red-cell (10^12^/L)	3.8–5.1	4.75	4.61		4.25	3.24	2.84	2.85	2.59	3.39
Hemoglobin (g/L)	115–150	145	143		130	98	86	85	78	101
Platelets (10^9^/L)	125–350	197	168		106	137	90	67	58	262
Neutrophil count (10^9^/L)	1.8–6.3	16.01	22.56		19.12	25.65	10.37	7.66	5.78	5.35
Neutrophil percentage (%)	40–75	93.3	93.61		96.24	95.9	91.31	86.21	86.5	72.11
*Renal function*										
Urea nitrogen (mmol/L)	2.9–7.2	4.6	7.6	9.9		10.6	11.3	8.9	7.2	8.2
Creatinine (umol/L)	53–132	52	117	124		146	155	137	116	112
Uric acid (umol/L)	150–360	180	223	239		321	378	296	312	393
*Liver function*										
Total protein (g/L)	66.0–83.0	60.5	56.2	55.2		36.2	42.3	45.8	50.2	73.9
Albumin (g/L)	40.0–55.0	34.5	36.8	33.8		24.3	30.3	33	34.4	47.1
Globulin (g/L)	20.0–40.0	26	19.4	21.4		11.9	12	22.8	15.8	26.8
*Thyroid hormone*										
Triiodothyronine (nmol/L)	1.23–3.08	0.638							0.582	1.365
Thyroxine (nmol/L)	65.64–181.47	64.37							78.99	94.287
Free triiodothyronine (pmol/L)	2.77–7.08	1.62							1.86	4.35
Free thyroxine (pmol/L)	11.97–21.88	10.26							14.84	18.14
Thyroid stimulating hormone (uIU/mL)	0.27–4.2	0.847							3.9	2.26
*Female hormones*										
Follicle stimulating hormone (mIU/mL)	4.6–8.6		4.77							6.96
Luteinizing hormone (IU/L)	1.5–7.0		3.75							7.64
Estradiol (pg/mL)	18–63		47							34
Testosterone (ng/mL)	0.15–0.51		2.13							0.24
Prolactin (ng/mL)	3.5–24.2		15.72							16.69
Dehydroepiandrosterone sulfate (ug/dL)	51–321		> 1000							167.2
Sex hormone binding globulin (nmol/L)			12.6							49.2
Cortisol (ug/dL)	8.7–22.4		> 60							12.44
*Cortisol rhythm*										
Cortisol (8 am) (ug/dL)	4.26–24.85		82.255					11.495		17.762
Cortisol (4 pm) (ug/dL)	1.9–17.3		92.705					5.847		6.107
Cortisol (12 pm) (ug/dL)			65.859					6.088		2.921
*ACTH rhythm*										
ACTH (8 am) (pg/mL)	7.2–63.3		231.756					36.489		34.742
ACTH (4 pm) (pg/mL)	3.6–31.7		242.763					30.08		25.388
ACTH (12 pm) (pg/mL)			202.883					22.947		35.454

### Imaging examinations

**Fig. 1:**
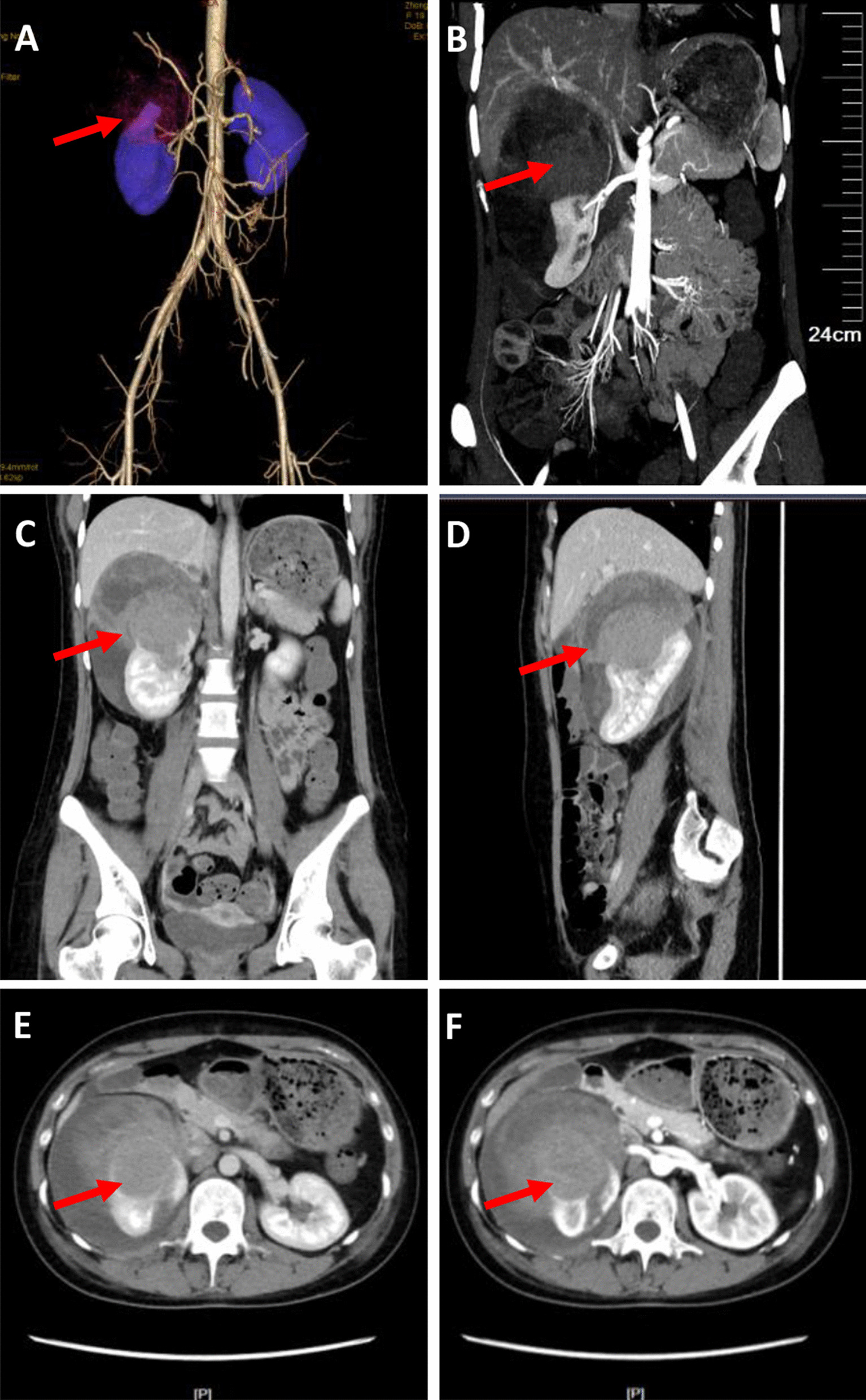
Abdominal imaging with contrast-enhanced computed tomography and computed tomography angiography images. **A**, **B** Computed tomography angiography images; **C**–**F** Abdominal imaging with contrast-enhanced computed tomography images

Abdominal imaging with contrast-enhanced computed tomography and computed tomography angiography (Sep 5) showed right upper pole occupancy (size 9.4 cm * 9.9 cm) with subepithelial hemorrhage, right renal artery branch supplied blood, and the right portal lymph node enlargement (Fig. 1).

### Treatment and follow-up

Re-examination of the patient’s blood routine, renal function and protein level showed that the infection index further increased, and the renal function and protein level decreased. The patient received double J tube implantation under left ureteroscope plus right radical nephrectomy plus clearance of right perirenal hematoma plus lysis of right perirenal adhesion under general anesthesiaon Sep 8. The patients were transferred to ICU monitoring and treatment after surgery. The blood routine, renal function and protein levels of the patients showed a trend of improvement for three consecutive days after surgery, ACTH and plasma total cortisol (PTC) decreased to normal, and thyroid hormones tended to normal. The patient’s facial swelling was reduced and the acne on her face and back subsided. Three days later the patient was transferred to the general ward and discharged on Sep 14. However, three weeks after surgery, the patient died while preparing for further chemotherapy.

### Pathology

**Fig. 2:**
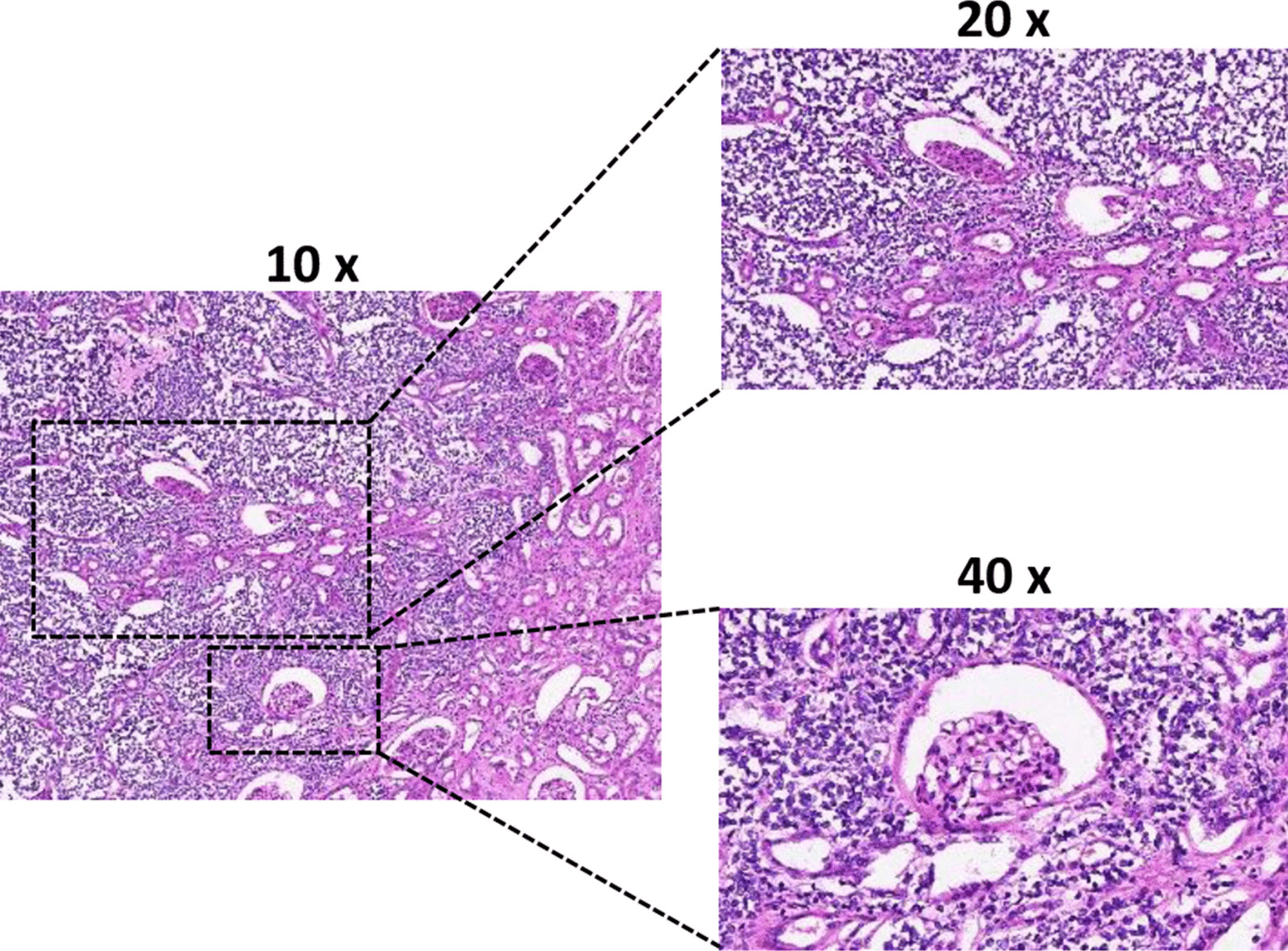
HE staining of tumor tissue

**Fig. 3:**
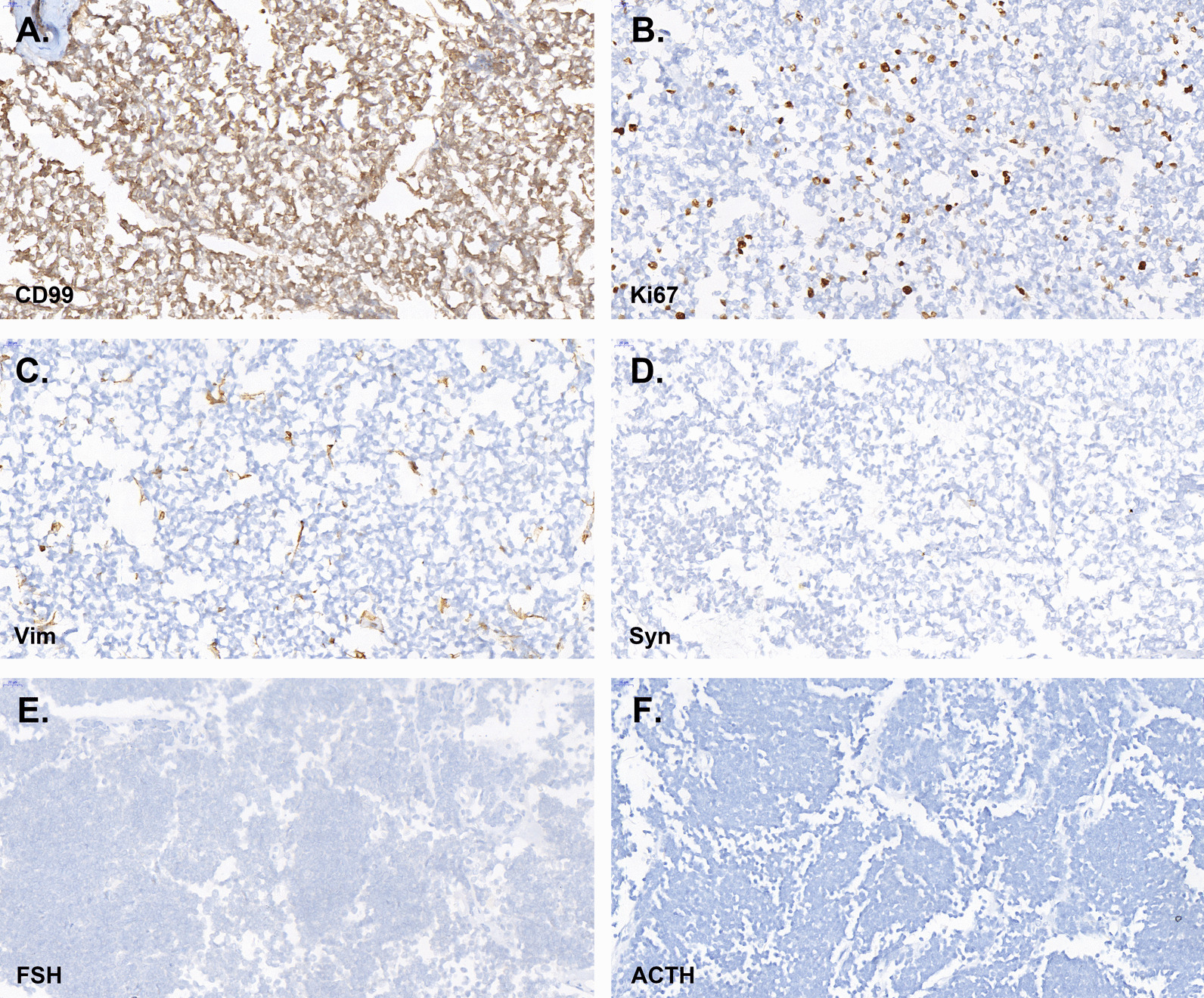
Immunohistochemical results in tumor tissue. **A** CD99; **B** Ki67; **C** Vim; **D** Syn; **E** FSH; **F** ACTH

**Fig. 4:**
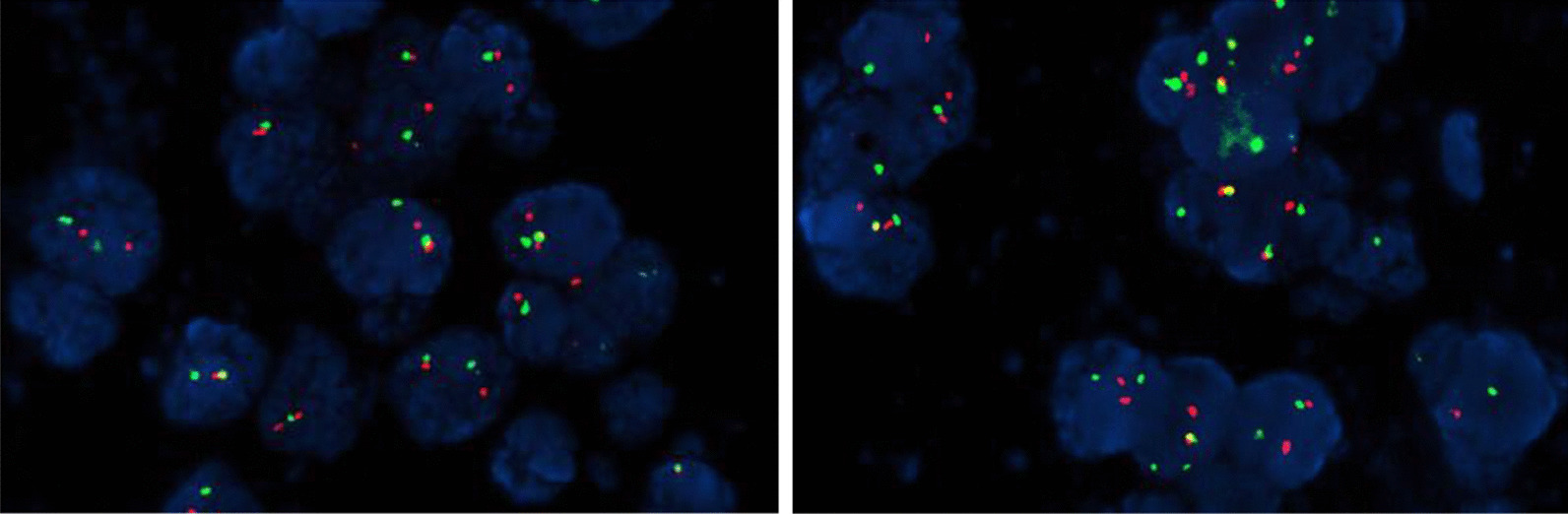
EWSRI gene fusion was detected by fluorescence in situ hybridization (FISH)

The maximum diameter of the tumor was 8.0 cm. The tumor involved renal parenchyma, perirenal adipose tissue and adrenal gland, and a carcinoma thrombus was seen in the vasculature. Under light microscope, the tumor cells were nested and sheet-like, composed of a large number of small round cells with relatively uniform morphology and little cytoplasm (Fig. 2). The immunohistochemical results were positive for CD99 (diffuse membranous positivity) and Ki67 (about 10%), while Vim, Syn, FSH and ACTH were negative (Fig. 3). EWSRI gene fusion was detected by fluorescence in situ hybridization (FISH) (Fig. 4). According to the results of postoperative pathology and molecular pathological examination, the finally diagnosed was renal small round blue cell tumor (consistent with PNET).

## Discussion and conclusions

PNET is a highly malignant small round blue cell tumor with neural differentiation. According to the location, PNET can be divided into central PNET (cPNET) and peripheral PNET (pPNET) [8]. Ewing sarcoma/PNET usually occurs in children and adolescents, and the most common site is bone or soft tissue in the trunk or axial bone. Ewing sarcoma/PNET in the genitourinary system is relatively rare, and even rarer in the kidney [9]. Renal Ewing sarcoma/PNET is a type of pPNET with a median age of about 27 years old, and is slightly more common in males [10].

The clinical manifestations of renal Ewing sarcoma/PNET are non-specific. The gross pathology of tumor tissue was gray, grayish brown, accompanied by hemorrhage, necrosis and cystic degeneration. In the histological examination of renal Ewing sarcoma/PNET, the tumor cells were arranged in nest shape, and Homer-Wright rosettes were seen. Homer-Wright rosettes are one of the main histological bases for the diagnosis of renal Ewing sarcoma/PNET [11]. The diagnosis of renal Ewing sarcoma/PNET mainly depends on pathological examination. With the improvement of immunohistochemical techniques, immunohistochemistry has become an important basis for pathological diagnosis of renal Ewing sarcoma/PNET [12]. CD99 is a monoclonal antibody that recognizes p30/32 glycoprotein and can be detected in almost all renal Ewing sarcoma/PNET [13]. In addition, some cases expressed vimentin, NSE, S-100 and Syn [14]. There is no standardized criteria for the pathological diagnosis of renal Ewing sarcoma/PNET, which is generally considered to be consistent with [15]: (1) Homer-Wright rosettes under light microscope; (2) positivity for CD99 and other neural markers (at least 2 kinds).

In recent years, it has been found that 90–95% of PNET have a t(11; 22) (q24; q12) chromosome translocation, leading to the production of the EWS/FLI-1 fusion gene [16]. It has been shown that the FISH method detects EWSR1 gene fusions with a sensitivity of 92.3% and a specificity of 100% [17]. Therefore, the FISH method to detect fusion genes formed by PNET-specific chromosomal translocations has greater diagnostic and differential diagnostic value. In summary, the diagnosis of renal Ewing sarcoma/PNET should be comprehensively judged by light microscopic morphology, immunohistochemistry and FISH method. In the pathological diagnosis of this patient, immunohistochemistry showed CD99 and Ki67 positivity, FISH showed EWSR1 gene fusion, and the diagnosis of small round blue cell tumor was confirmed.

According to previous reports, ectopic ACTH syndrome is primarily associated with small cell carcinomas and carcinoid tumors, and also be associated with mediastinum, pancreases, thymus, and pheochromocytoma [18–20]. Neuroendocrine tumors rarely produce excessive ACTH and cause ectopic ACTH syndrome. Shimizu et al. [21] reported a case of renal PNET accompanied by elevated plasma ACTH levels. We describe a case of ectopic ACTH syndrome and hypothyroidism caused by a renal Ewing sarcoma/PNET. Although our patient received radical nephrectomy treatment, postoperative ACTH and PTC decreased to normal, and thyroid hormones tended to normalize. The patient's facial swelling was alleviated and the acne on the face and back subsided. However, due to the highly malignant and aggressive nature of the tumor, the patient died in preparation for further chemotherapy.

Renal Ewing sarcoma/PNET is more prone to recurrence and metastasis than other renal tumors, with the most common site of metastasis being the lung, followed by the liver and bone [22]. 1/3 of patients have a renal vein or inferior vena cava thrombus at the time of diagnosis, which makes treatment often ineffective. The prognosis of renal Ewing sarcoma/PNET is generally poor, with a 5-year overall survival rate of approximately 45–55% [5]. Due to the rarity of renal Ewing sarcoma/PNET, there is no uniform standard of treatment for renal Ewing sarcoma/PNET, and treatment is based on a combination of surgical resection with adjuvant radiotherapy and targeted therapy. The best surgical procedure is radical nephrectomy. Chemotherapy can improve the prognosis of the disease, and a multidrug chemotherapy regimen is recommended. The most effective regimen is a combination of vincristine, cyclophosphamide, adriamycin, etoposide, and isocyclophosphamide. Renal Ewing sarcoma/PNET is still a rare tumor, and more cases need to be accumulated to explore better treatment options.

## Data Availability

The dataset used during the study are available from the corresponding author on a reasonable request.

## Abbreviations

PNET: Primitive neuroectodermal tumor
PTC: Plasma total cortisol
ACTH: Adrenocorticotrophic hormone
CT: Computed tomography

## References

[1] Watson S, Perrin V, Guillemot D, Reynaud S, Coindre JM, Karanian M, Guinebretiere JM, Freneaux P, Le Loarer F, Bouvet M (2018). Transcriptomic definition of molecular subgroups of small round cell sarcomas. J Pathol.

[2] Gupta S, Billadello L, Casalino DD (2012). Renal primitive neuroectodermal tumor. J Urol.

[3] Shibui Y, Miyoshi K, Kohashi K, Kinoshita Y, Kuda M, Yamamoto H, Taguchi T, Oda Y (2019). Glypican-3 expression in malignant small round cell tumors. Oncol Lett.

[4] Cohn SL (1999). Diagnosis and classification of the small round-cell tumors of childhood. Am J Pathol.

[5] Hamidi N, Esen B, Kivrak H, Sertcelik A, Gulpinar O (2015). A large and metastatic primitive neuroectodermal tumor of the kidney. Turk J Urol.

[6] Terzolo M, Reimondo G, Ali A, Bovio S, Daffara F, Paccotti P, Angeli A (2001). Ectopic ACTH syndrome: molecular bases and clinical heterogeneity. Ann Oncol.

[7] Comi RJ, Gorden P (1998). Long-term medical treatment of ectopic ACTH syndrome. South Med J.

[8] Batsakis JG, Elnaggar AK (1997). Ewingʼs sarcoma and primitive neuroectodermal tumors: cytogenetic cynosures seeking a common histogenesis. Adv Anat Pathol.

[9] Mohsin R, Hashmi A, Mubarak M, Sultan G, Shehzad A, Qayum A, Naqvi SA, Rizvi SA (2011). Primitive neuroectodermal tumor/Ewing's sarcoma in adult uro-oncology: a case series from a developing country. Urol Ann.

[10] Aghili M, Rafiei E, Mojahed M, Zare M (2012). Renal primitive neuroectodermal tumor: does age at diagnosis impact outcomes?. Rare Tumors.

[11] Song HC, Sun N, Zhang WP, Huang CR (2012). Primary Ewing's sarcoma/primitive neuroectodermal tumor of the urogenital tract in children. Chin Med J (Engl).

[12] Patnaik N, Mishra K, Saini P, Agarwal N (2015). Primitive neuroectodermal tumor of the kidney in a young male: case report and review of literature. Urol Ann.

[13] Nerli RB, Hiremath MB, Prabha V, Malur P, Borges A (2010). Primitive neuroectodermal tumor (PNET) of the kidney with level IV inferior vena caval thrombus: a case report. Recent Res Sci Technol.

[14] Karpate A, Menon S, Basak R, Yuvaraja TB, Tongaonkar HB, Desai SB (2012). Ewing sarcoma/primitive neuroectodermal tumor of the kidney: clinicopathologic analysis of 34 cases. Ann Diagn Pathol.

[15] Folpe AL, Hill CE, Parham DM, O'Shea PA, Weiss SW (2000). Immunohistochemical detection of FLI-1 protein expression: a study of 132 round cell tumors with emphasis on CD99-positive mimics of Ewing's sarcoma/primitive neuroectodermal tumor. Am J Surg Pathol.

[16] Wu Y, Zhu Y, Chen H, Huang Y, Wei Q, Chen H, Xie X, Li X, Zhou Q, Yang Y (2010). Primitive neuroectodermal tumor of the kidney with inferior vena cava tumor thrombus during pregnancy response to sorafenib. Chin Med J.

[17] Rekhi B, Vogel U, Basak R, Desai SB, Jambhekar NA (2014). Clinicopathological and molecular spectrum of ewing sarcomas/PNETs, including validation of ewsr1 rearrangement by conventional and array FISH technique in certain cases. Pathol Oncol Res.

[18] Cieszyński Ł, Obołończyk MB, Szulc M, Sworczak K (2016). Cushing's syndrome due to ectopic ACTH secretion. J Clin Endocrinol Metab.

[19] Levine AC, Sanchez J (2017). Commentary on cushing syndrome due to ectopic acth. Endocr Pract.

[20] Ilias I, Torpy DJ, Pacak K, Mullen N, Wesley R, Nieman LK (2005). Cushing’s syndrome due to ectopic corticotropin secretion: twenty years’ experience at the National Institutes of Health. J Clin Endocrinol Metab.

[21] Shimizu N, Hasumi M, Hamano T, Iijima M, Yoshioka T, Yamazaki Y, Sasano H (2019). Renal primitive neuroectodermal tumor with elevated plasma adrenocorticotropic hormone levels: a case report. IJU Case Rep.

[22] Hakky TS, Gonzalvo AA, Lockhart JL, Rodriguez AR (2013). Primary Ewing sarcoma of the kidney: a symptomatic presentation and review of the literature. Ther Adv Urol.

